# Quantifying blood-brain barrier leakage in small vessel disease: Review and consensus recommendations

**DOI:** 10.1016/j.jalz.2019.01.013

**Published:** 2019-06

**Authors:** Michael J. Thrippleton, Walter H. Backes, Steven Sourbron, Michael Ingrisch, Matthias J.P. van Osch, Martin Dichgans, Franz Fazekas, Stefan Ropele, Richard Frayne, Robert J. van Oostenbrugge, Eric E. Smith, Joanna M. Wardlaw

**Affiliations:** a Centre for Clinical Brain Science, University of Edinburgh, Edinburgh, UK; b Dementia Research Institute, University of Edinburgh, Edinburgh, UK; c Edinburgh Imaging, University of Edinburgh, Edinburgh, UK; d Department of Radiology & Nuclear Medicine, School for Mental Health and Neuroscience, Maastricht University Medical Centre, Maastricht, The Netherlands; e Leeds Imaging Biomarkers group, Department of Biomedical Imaging Sciences, University of Leeds, Leeds, UK; f Department of Radiology, Ludwig-Maximilians-University Hospital Munich, Munich, Germany; g Department of Radiology, C. J. Gorter Center for High Field MRI, Leiden University Medical Center, Leiden, The Netherlands; h Institute for Stroke and Dementia Research, University Hospital, Ludwig-Maximilians-University München & Munich Cluster for Systems Neurology (SyNergy), Munich, Germany; i Department of Neurology, Medical University of Graz, Graz, Austria; j Department of Radiology, Hotchkiss Brain Institute, University of Calgary, Calgary, Alberta, Canada; k Department of Clinical Neurosciences, Hotchkiss Brain Institute, University of Calgary, Calgary, Alberta, Canada; l Seaman Family MR Research Centre, Foothills Medical Centre, Calgary, Alberta, Canada; m Department of Neurology, School for Mental Health and Neuroscience, Maastricht University Medical Centre, Maastricht, The Netherlands

**Keywords:** Cerebral small vessel disease, Dementia, Blood-brain barrier, Endothelial dysfunction, DCE-MRI, Permeability, MRI

## Abstract

Cerebral small vessel disease (cSVD) comprises pathological processes of the small vessels in the brain that may manifest clinically as stroke, cognitive impairment, dementia, or gait disturbance. It is generally accepted that endothelial dysfunction, including blood-brain barrier (BBB) failure, is pivotal in the pathophysiology. Recent years have seen increasing use of imaging, primarily dynamic contrast-enhanced magnetic resonance imaging, to assess BBB leakage, but there is considerable variability in the approaches and findings reported in the literature. Although dynamic contrast-enhanced magnetic resonance imaging is well established, challenges emerge in cSVD because of the subtle nature of BBB impairment. The purpose of this work, authored by members of the HARNESS Initiative, is to provide an in-depth review and position statement on magnetic resonance imaging measurement of subtle BBB leakage in clinical research studies, with aspects requiring further research identified. We further aim to provide information and consensus recommendations for new investigators wishing to study BBB failure in cSVD and dementia.

## 1 Introduction

*Cerebral small vessel disease (cSVD)* is an umbrella term that covers all pathological processes of the small vessels in the brain [1]. The most common form is age-associated and vascular risk factor–associated microangiopathy, which may manifest as acute symptoms (lacunar stroke), as slowly progressive symptoms, including cognitive impairment and gait disturbances, and in magnetic resonance imaging (MRI)–visible structural brain changes, such as white matter hyperintensities (WMHs), enlarged perivascular spaces, small subcortical infarcts, and cerebral microbleeds [2]. It is generally accepted that endothelial dysfunction plays a pivotal role in the early development of cSVD, and there is consequently growing interest in the use of advanced neuroimaging methods to provide quantitative functional information such as cerebrovascular reactivity, cerebral blood flow (CBF) and pulsatility, and blood-brain barrier (BBB) integrity [3]. Such techniques provide quantitative information on changes of the cerebrovascular system including the microvasculature that may predate the subsequent emergence of classic radiological and clinical signs of cSVD and are therefore particularly relevant for mechanistic studies and as specific endpoints in clinical trials of drugs with relevant modes of action, for example, those with effects on endothelial function [4].

**Fig. 1:**
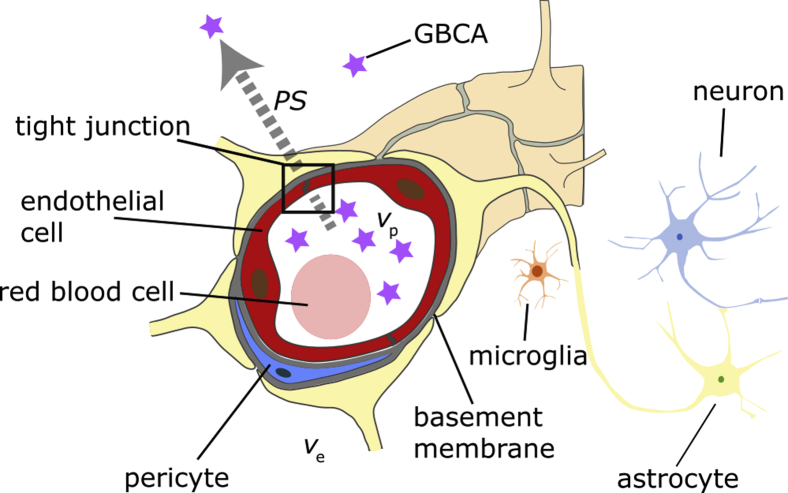
Schematic diagram showing the neurovascular unit. Leakage of gadolinium-based contrast agent (GBCA) molecules across the blood-brain barrier, from the capillary blood plasma space (volume fraction *v*_p_) to the extravascular extracellular space (volume fraction *v*_e_), is illustrated by the arrow. The rate of leakage per unit tissue volume and per unit capillary blood plasma GBCA concentration is described by the permeability–surface area product (*PS*).

Although evidence is presently limited, several studies of patients with small subcortical infarcts or vascular cognitive impairment due to cSVD have suggested that slightly increased BBB leakage is associated with clinical or imaging features of cSVD. This points to an important potential pathophysiologic role for BBB failure in the development of brain tissue damage and the progression of these features over time [1]. BBB integrity is therefore an important target for assessment in studies of pathophysiology and could have an important role in the evaluation of treatment. However, the supposed elevation in BBB permeability associated with cSVD, aging, or dementia is expected in general to be very subtle and is therefore much more difficult to capture than the gross disruption of the BBB seen with acute inflammation, neoplasms, or infarction. Unlike these other pathologies, where signal changes are conspicuous on *T*_1_-weighted (*T*_1_w) or fluid-attenuated inversion recovery (FLAIR) scans after administration of gadolinium-based contrast agents (GBCAs), the effect of cSVD-related BBB degradation on post-contrast MRI signal changes is smaller and will therefore likely benefit from the application of sophisticated quantification methods. The structure of the BBB and slow leakage of GBCAs are illustrated schematically in Fig. 1.

**Fig. 2:**
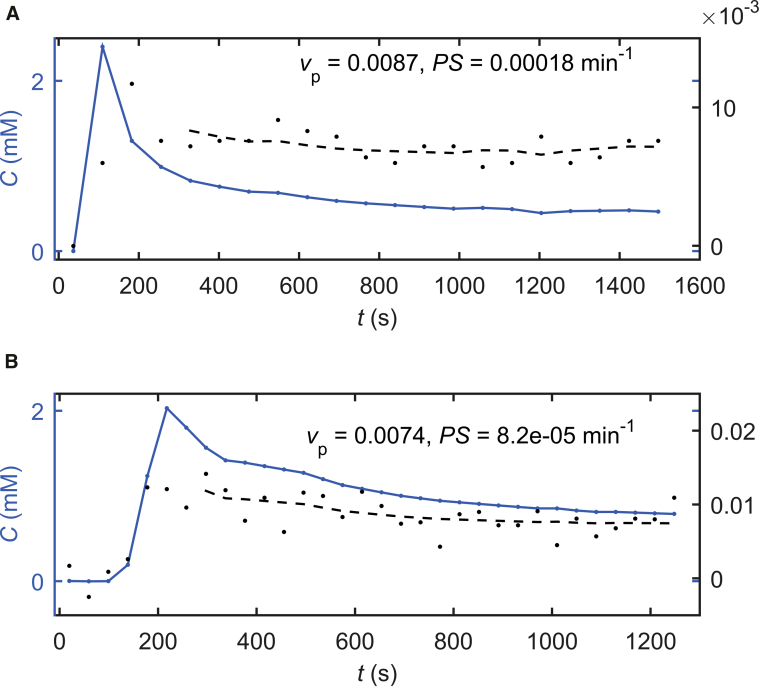
Illustrative dynamic contrast-enhanced magnetic resonance imaging (DCE-MRI) data in two patients with cerebral small vessel disease (cSVD) with a history of nondisabling stroke showing estimated concentrations of gadolinium-based contrast agent (GBCA) in blood plasma (*c*_p_, blue), white matter (*C*_t_, black), and the fitted Patlak model (dashed line). Data were acquired and processed by the authors using the following protocols: (A) 1.5-T MRI with bolus injection of 0.1 mmol/kg gadoteric acid and a three-dimensional spoiled gradient echo (sGRE) sequence (acquired spatial resolution 0.94 × 1.25 × 4 mm, temporal resolution 73 s, post-injection acquisition time 24 min) and variable flip angle *T*_1_ measurement; the median signal from a semiautomatically generated normal-appearing white matter mask was fitted [7]. (B) 3-T MRI with 3-minute slow injection of 0.1 mmol/kg gadobutrol, 3D sGRE (acquired spatial resolution 2 mm isotropic, temporal resolution 40 s, DCE-MRI acquisition time 21 minutes), and *T*_1_ and flip angle measurement via the DESPOT1-HIFI method; the mean white-matter signal from a region drawn manually in the centrum semiovale was modeled. Blood GBCA concentration (“vascular input function” [VIF]) was sampled in the superior sagittal sinus. The derived Patlak model parameters *v*_P_ and *PS* represent the capillary blood plasma volume fraction and the permeability–surface area product, respectively.

For quantitative measurement, *dynamic contrast-enhanced MRI* (DCE-MRI) has been the imaging technique of choice for assessing BBB failure in cSVD and in other low-permeability applications [5], [6]. In this method, the slow accumulation of paramagnetic GBCAs in the extracellular extravascular space (EES) is detected via the *T*_1_-shortening effect on tissue water. As shown in Fig. 2, the vascular and extravascular contributions to the signal enhancement can be separated by measuring the GBCA concentration in both a vessel (“vascular input function [VIF]”) and the tissue; the tissue concentration can then be fitted using a pharmacokinetic model to separate the vascular and extravascular components. Careful application of this approach can yield quantitative estimates of the BBB leakage rate (per unit volume and blood plasma GBCA concentration), the blood plasma volume fraction, and other physiological measures [8].

The HARNESS (HARmoNising Brain Imaging MEthodS for VaScular Contributions to Neurodegeneration; www.harness-neuroimaging.org) Initiative was formed in 2016, funded by the multinational Joint Programme–Neurodegenerative Disease Research (JPND). The objective of HARNESS is to standardize and disseminate neuroimaging acquisition and analysis protocols for measuring the contributions of vascular disease to dementia and neurodegeneration [9]. As a component of this initiative, a subgroup convened on four occasions in 2017 to consider emerging techniques for BBB leakage imaging, comprising experts in magnetic resonance (MR) physics, neuroradiology, and neurology. This article reflects the outcome of these meetings, and it will focus on the DCE-MRI approach as being, currently, the most evolved and promising technique for obtaining quantitative, local estimates of BBB leakage in brain tissue. We also briefly address other imaging and nonimaging approaches to assess BBB integrity, such as MRI quantification of water exchange rates and biochemical methods, noting that some of these may deserve greater prominence in future following further technical development and validation. Although cSVD is the clinical focus of this work, we have referred to technically relevant work in other diseases. In Section 2, we summarize applications in cSVD and assess the technical progress of this potential imaging biomarker (IB). In Section 3, we then provide detailed explanations of the image acquisition and data analysis steps, highlighting the diversity of approaches taken and the challenges and uncertainties associated with each. Where possible, we provide recommendations for obtaining BBB leakage measurements in future clinical studies of cSVD and dementia as a step toward standardization. Where possible, these are based on evidence from the literature. Where sufficient good-quality evidence is lacking, we aimed to put forward consensus-based recommendations as a starting point for further development and to reduce heterogeneity in the future literature. We also identify aspects of data acquisition and analysis procedures for which there is insufficient evidence to provide firm recommendations and where further basic methodological research is needed. In Section 4, we address practical aspects of imaging cognitively impaired patients. Finally, in Section 5, we locate the technique within an IB framework [10], identifying three priority areas for future development.

## 2 Summary of clinical studies and methods

### 2.1 DCE-MRI studies in cSVD

DCE-MRI has been used to assess BBB permeability effects in several cSVD and other related conditions, including vascular cognitive impairment [11], [12], Binswanger's disease [13], [14], cognitive impairment and dementia [11], [12], [13], [15], [16], [17], [18], [19], [20], small-vessel stroke and minor stroke [7], [12], [14], [18], [21], [22], [23], [24], [25], [26], type 2 diabetes [27], and aging [17]. An overview of the acquisition and analysis methods used in clinical studies and their findings in relation to cSVD is provided in Table 1.

**Table 1:** Summary of methods and clinical findings from DCE-MRI studies of cSVD

First author (year)	Participants (*n*)	Contrast agent	Acquisition	Processing	Main findings
Bronge (2000) [20]	Dementia/cognitively impaired with WMH (10)	0.2 mmol/kg gadodiamide	1.5 T; spin-echo and sGRE; Δ*t* = 10 min; *TA* = 30 min	Change in signal and WMH/NAWM signal ratio	No increase in either variable after contrast injection
Hanyu (2002) [14]	BD (17), minor stroke (10), age-matched controls (14)	0.1 mmol/kg gadopentetic acid	1.5 T; pre-injection and 15-min-post-injection 2 × TR fast spin-echo *T*_1_ mapping	Relative *T*_1_ change	Greater *T*_1_ reduction in WMH in BD than NAWM in controls or WMH in minor stroke; positive association with cognition in BD patients
Wang (2006) [19]	MCI (11), age-matched controls (11)	15 s manual injection 1 mL/10 lb gadodiamide	1.5 T; sGRE; Δ*t* = 25.5 s; *TA* = 5 min	“BBB permeability index” derived from late enhancement	No significant intergroup differences; no association with age or cognition
Wardlaw (2008) [25] Wardlaw (2009) [23] Wardlaw (2013) [24]	Lacunar or mild cortical ischemic stroke (100)	40 mL bolus gadodiamide	1.5 T; sGRE; Δ*t* = 69 s; *TA* = 30 min	Mixed linear model of signal enhancement	Enhancement greater in lacunar versus cortical stroke in WM and CSF; association (in basal ganglia) with worse outcome
Starr (2009) [15]	AD (15), healthy older people (15)	20 mL bolus gadopentetic acid	1.5 T sGRE; Δ*t* = 3 min 46 s; *TA* = 30 min	Mixed linear model of signal	No significant difference between groups; significant time-AD interaction effect on signal
Topakian (2010) [22]	Lacunar syndrome with MRI infarct (28), controls (21)	40 mL gadodiamide bolus	1.5 T; sGRE; Δ*t* = 65 s; *TA* = 28 min	Area under signal enhancement curve	Greater AUC in the cSVD group in NAWM; WMH burden predicts AUC in NAWM and CSF
Israeli (2011) [28]	Ischemic stroke (34)	Not specified	3 T; spin-echo T1w; Δ*t* = 7 min; *TA* = 14 min	Images and subtraction maps used to calculate “BBB opening score”	Lower BBB opening score in lacunar versus nonlacunar stroke lesions
Taheri (2011) [11]	VCI (60), controls (20)	0.025 mmol/kg bolus gadopentetic acid	1.5 T; serial *T*_1_ mapping (TAPIR); Δ*t* = 3.5 min; *TA* = 25 min	Patlak model; VIF in SSS	WM *K*_i_ higher versus controls; no association with age or CSF albumin
Huisa (2015) [13]	VCI with BD (22), age-matched controls (16)	0.025 mmol/kg bolus gadopentetic acid	1.5 T, 3 T; serial *T*_1_ mapping (TAPIR); Δ*t* = 2.5–3.5 min; *TA* = 22.5–24.5 min	Patlak model with threshold to generate “abnormal WMP”	WMP higher versus controls; no correlation with WMH load; minimal overlap of WMP regions at initial and follow-up scans
Montagne (2015) [17]	NCI (24), MCI (21)	0.05 mmol/kg gadobenic acid	3 T; sGRE *T*_1_ mapping and dynamic scan; Δ*t* = 15.4 s; *TA* = 16 min	Patlak model; VIF in common carotid artery	Age-dependent increase in hippocampal *K*^Trans^ among NCI only; hippocampal *K*^Trans^, CSF/plasma albumin ratio, and sPDGFRβ higher in MCI
Heye (2016) [7]	Lacunar or mild cortical ischemic stroke (264)	0.1 mmol/kg bolus gadoteric acid	1.5 T; sGRE *T*_1_ mapping and dynamic scan; Δ*t* = 73 s; *TA* = 24 min	Patlak model; VIF in SSS	*K*^Trans^ greater in WMH versus NAWM
Munoz Maniega (2017) [21]				Signal enhancement slope	Signal enhancement slope in WM increases with WMH burden
Wardlaw (2017) [26]				Mixed linear model of signal enhancement with time interaction terms	Slope in NAWM increases with age and WMH burden
van de Haar (2016) [29] van de Haar (2016) [30]	Early AD (16), controls (18)	0.1 mmol/kg bolus gadobutrol	3 T; SR-sGRE *T*_1_ mapping and dual temporal resolution dynamic scan; Δ*t* = 3.2/31.8 s; *TA* = 25 min	Patlak model; VIF in SSS; histogram analysis to estimate “leakage volume” (*v*_L_)	Higher GM *K*_i_ and *v*_L_ in AD, negatively associated with CBF in patients
Zhang (2017) [12] Zhang (2018) [18]	mVCI and lacunar stroke (80), age-/sex-matched controls (40)	0.1 mmol/kg bolus gadobutrol	3 T; SR-sGRE *T*_1_ mapping and dual temporal resolution dynamic scan; Δ*t* = 3.2/30.5 s; *TA* = 24 min	Patlak model; VIF in SSS; histogram analysis to estimate *v*_L_	*v*_L_ but not *K*_i_ higher in cSVD; lower *K*_i_, higher *v*_L_ in WMH associated with WMH volume but not cognition in cSVD; *K*_i_ in GM and NAWM not associated with WMH volume.
Li (2018) [31]	Participants presenting to neurology department (diseases/symptoms not specified; 99)	0.1 mmol/kg bolus unspecified GBCA	3 T; sGRE *T*_1_ mapping and dynamic scan; Δ*t* = 3.8 s; *TA* = 3.5 min	Patlak model; VIF in SSS	*K*^Trans^ positively associated with cSVD burden and individual cSVD features

Abbreviations: AD, Alzheimer's disease; AUC, area under curve; BBB, blood-brain barrier; BD, Binswanger disease; CSF, cerebrospinal fluid; cSVD, cerebral small vessel disease; DCE-MRI, dynamic contrast-enhanced magnetic resonance imaging; Δ*t*, temporal resolution; GBCA, gadolinium-based contrast agent; GM, gray matter; *K*_i_=*K*^Trans^/(1 − Hct); *K*^Trans^, volume transfer constant; mVCI, mild vascular cognitive impairment; NAWM, normal-appearing white matter; NCI, no cognitive impairment; *PS*, permeability–surface area product; sPDGFRβ, soluble platelet-derived growth factor receptor β; sGRE, spoiled gradient echo; SR, saturation recovery; SSS, superior sagittal sinus; *TA*, DCE-MRI acquisition duration; TAPIR, *T*_1_ mapping sequence with partial inversion recovery; VCI: vascular cognitive impairment; VIF, vascular input function; WM, white matter; WMH, white matter hyperintensity; WMP, white matter permeability.

Some studies assessed the relationship between cSVD and BBB leakage by comparing patients with lacunar (i.e., small vessel) stroke and those with cortical stroke, reporting variously lower [26] and higher [23], [25] BBB leakage in the white matter (WM) and greater leakage of GBCA into the cerebrospinal fluid (CSF) [23], [26]. BBB leakage has also been studied in relation to WMH burden or total cSVD score [32] as indicators of disease burden, with some studies showing a positive association [21], [22], [26], [31] between BBB leakage and disease burden and other studies reporting negative or nonsignificant associations between WMH volume and leakage in various brain tissue types [13], [18]. Some studies compared patients with cSVD with controls, reporting greater leakage in the disease groups [11], [13], [14], [17], [22], [30] and, in two studies, no significant difference in the leakage rate [12], [19]. One study reported an association between BBB leakage and worse functional outcome at long-term follow-up [24].

Many studies reported leakage measures in both normal-appearing WM and WMH, although few reported on whether the difference was statistically significant. One study did report significantly greater leakage in WMH versus normal-appearing WM [7], including greater leakage with increasing proximity to the WMH [26], whereas another study reported no significant difference [20]; other studies quoted lower [12] and higher [14], [30], [31] leakage rates in WMH versus normal-appearing WM.

Although there is a degree of convergence in the literature regarding the relevance of BBB permeability in cSVD, there is, as described above and in Table 1, significant variation in both methodology and results, including order-of-magnitude differences in reported leakage rates [16]. A number of effects may explain the variation in reported findings. Some of these are pertinent to clinical studies in general, including sample size considerations (*n* = 10–264 patients), study design, and differing approaches to statistical analysis and correction for risk factors and other variables. For cSVD studies, participants are recruited through a variety of pathways and from various populations, including patients presenting with cognitive impairment, acute stroke, or dementia; participants may be in various stages of the disease process.

Importantly, the lack of a common approach to measuring BBB failure by MRI presents a substantial additional barrier to comparison and interpretation of the data. A wide range of acquisition protocols, analysis techniques, and “leakage” or “permeability” metrics (Table 1) have been used in these studies. To an extent, these approaches may represent different measures of the same underlying physiology and of different aspects of BBB function; however, some measurements may be strongly confounded by other biological and instrumental factors and may therefore be inadequate indicators of BBB integrity. Fortunately, there has been moderate but significant methodological progress over the past several years, with a small number of publications beginning to identify and address the technical limitations [7], [33], [34], [35], [36], which, while somewhat well known within the DCE-MRI field, can be particularly salient in the situations of subtle vessel permeability considered here. These limitations, the diversity of approaches noted above, and steps toward more targeted and harmonized IBs of subtle BBB failure are discussed in the following sections.

### 2.2 Alternative methods for assessing BBB leakage

Several alternative imaging-based methods for detecting subtle BBB leakage have been proposed. Dynamic susceptibility contrast (DSC-) MRI (“perfusion MRI” or “first-pass perfusion MRI”) has been proposed for characterizing BBB leakage in oncology [37] and stroke [38], [39], [40]. However, the typically short acquisition time, the difficulty of modeling the effects of contrast agent on both *T*_2_* and *T*_*1*_, which have a strong dependence on microstructural properties such as vessel size, and the challenge of disentangling perfusion and leakage effects make quantitative evaluation with DSC-MRI particularly challenging in cSVD, where the leakage rate is typically orders of magnitude lower than in tumors [41], [42].

Multicompartment modeling of the arterial spin labeling signal provides a potential route to measuring BBB permeability to water by separating the intravascular and extravascular contributions based on the different diffusion [43], [44], [45] or transverse relaxation [46], [47], [48] properties of the two compartments. A method for estimating global water BBB permeability has also been recently proposed, derived from the arterial spin labeling signal measured in veins [49]. Arterial spin labeling–based methods have the important advantage of not requiring GBCA administration, but sensitivity limitations are currently a barrier to reliable measurement in gray matter (GM) and especially in WM. Water exchange dynamics can also be probed via their effect on the spoiled gradient echo (sGRE) signal after GBCA administration [50]. However, the relationship between water permeability and BBB integrity, in the sense of the BBB's protective function, is uncertain. Abnormal water permeability may in fact signify distinct and multiple physiological aspects such as aquaporin function and metabolic turnover. Finally, GBCA-induced enhancement of CSF on *T*_2_w-FLAIR and *T*_1_w images allows detection of leakage through the BBB or blood-CSF barrier [23], [51]. This approach provides qualitative leakage information, not tissue leakage rates, but may be straightforward to use as a marker of leakage that may be clearly visible using standard structural sequences. Presently, it remains unclear through which route pericortical CSF enhancement is achieved, whether through BBB defects via perivascular spaces [23] and thence to the ventricles or cortical surface, or via defects in the blood-CSF barrier, for instance in the choroid plexus, or via both pathways. Such measurements have been performed hours or even days after administration of contrast [52], [53], which illustrates the slow nature of the leakage and may suggest that in practice, imaging does not need to be performed immediately after contrast administration.

Subtle leakage has also been measured previously using the ^68^Ga ethylenediaminetetraacetic acid (^68^Ga EDTA) tracer with positron emission tomography [54], [55], [56], [57], [58], but the use of ionizing radiation, high costs, lack of available infrastructure, and limited spatial resolution have limited their use in cSVD research. Computed tomography methods have also been reported [59], [60], but these approaches also carry risk from radiation dose and require iodinate contrast agent administration. A key advantage of MRI-based approaches is the opportunity to additionally characterize a range of structural cSVD features with high resolution and contrast-to-noise ratio (CNR) to assess the patient cSVD burden and to segment relevant regions of interest for BBB leakage measurement.

Many nonimaging studies have assessed BBB disruption in normal aging, dementia, and vascular disease using biochemical methods in CSF or plasma [5], [61], primarily via the CSF/serum albumin ratio. However, such global markers, as well as being invasive, do not provide information on the rate, anatomical locations, or tissue classes associated with BBB leakage.

### 2.3 Accuracy and reproducibility

Owing to the lack of reliable, convenient reference methods, there have been few attempts to validate or compare DCE-MRI measurements of subtle leakage against other techniques in humans. Taheri et al. reported a significantly higher CSF albumin index and higher leakage rate measured by DCE-MRI among cognitively impaired patients with “suspected microvascular disease with extensive WM involvement” versus controls but did not find a significant correlation between the two measures [11]. Montagne et al. reported a correlation between both the CSF/plasma albumin ratio and a marker of pericyte dysfunction (CSF platelet–derived growth factor receptor β) with DCE-MRI leakage rates in the hippocampus among two age-matched groups (combined) of participants with mild and no cognitive impairment respectively; however, significant associations with leakage rates measured in other brain regions were not reported and the effects of partial volume artifact in these small regions of interest and of inflow effect on the VIF are unknown [17]. Additional corroborative data between blood, CSF, or histological (e.g. Evans Blue, tissue fibrinogen, Claudin-5, or immunoglobulin G) markers of BBB failure and DCE-MRI in humans or in rodent models or validation using custom-built phantoms with appropriate hemodynamic and permeability characteristics would permit greater confidence in the technique. Despite the near absence of such validation, sources of systematic errors in such measurements in humans have been explored and will be discussed in the following sections [7], [33], [35], [62].

Further information on reproducibility would also aid development as a quantitative IB but requires repeated administration of GBCA and is likely to depend on the acquisition and processing methods used. We are aware of only one reproducibility study reported in the literature to date [36], which found, among a group of patients with mixed cerebrovascular diseases scanned at 3 T, coefficients of variation of 11.6 % and 14.4 % for WM and GM leakage rates, respectively.

## 3 Review of methodology and HARNESS recommendations

**Fig. 3:**
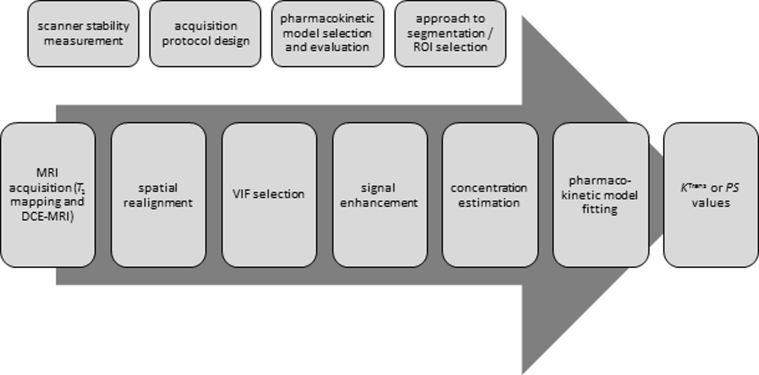
Schematic block diagram illustrating the steps required to quantify subtle BBB leakage of GBCA. The steps indicated above the arrow are performed during the pilot phase or as part of quality assurance procedures. Abbreviations: BBB, Blood-brain barrier; DCE-MRI, dynamic contrast-enhanced magnetic resonance imaging; GBCA, gadolinium-based contrast agent; *K*^Trans^, volume transfer constant; *PS*, permeability–surface area product; VIF, vascular input function.

In the following sections, we review key aspects of subtle BBB leakage measurement by DCE-MRI, including the main challenges, uncertainties, and pitfalls associated with each acquisition and processing step (summarized in Fig. 3). Where possible, we provide consensus recommendations for current practice and identify aspects where further primary research is needed to support future recommendations—these recommendations are summarized in Table 2.

**Table 2:** Summary of the main HARNESS consensus recommendations for implementation and future development of BBB leakage imaging

Category	Recommendations	Research questions and objectives
MRI hardware	• 1.5 T or 3 T • Head coil with high sensitivity and homogeneity • Maximal temporal stability in signal and *B*_1_ • Padding to restrict head motion • QA program including “sham” DCE-MRI without contrast in volunteers	• Influence of field strength on precision • Application/development of motion reduction/compensation techniques
Pulse sequence	• 3D spoiled gradient echo or 3D saturation-recovery spoiled gradient echo • Reliable pre-injection *T*_1_ measurement	• Effect on precision • Influence of flip angle inhomogeneity • Artifact reduction
Acquisition parameters	• Spatial resolution sufficient to determine VIF and smallest structures of interest without partial volume artifact • Temporal resolution 1 min or better • 15-20 minute DCE-MRI acquisition time	• Influence of sequence parameters and injection protocol on precision and accuracy
Contrast agent	• Standard dose of low–molecular weight GBCA • Selection based on latest appropriate (e.g., EMA/FDA) safety guidance	• Novel, safe contrast agents with greater *T*_1_ relaxivity and BBB permeability • Development and validation of non-exogenous contrast methods
Preprocessing	• Spatial realignment of time series • Signal-concentration conversion, using pre-contrast *T*_1_ measurement if available	• Utility of flip angle correction • Influence of water exchange rates • Tissue dependence of relaxivity
Pharmacokinetic modeling	• Fit time-concentration data to appropriate pharmacokinetic (typically Patlak) model	• Causes and correction of signal drift • Spatiotemporal noise structure
Vascular input function	• Measurement of individual patient VIF in a large venous sinus	• Evaluation of signal phase for estimating VIF
Regional measurement	• Report representative *PS* (*K*^Trans^) measurements for each region or tissue • Minimize cross-contamination between tissues due to partial volume artifact and image misregistration	• Development and validation of postprocessing methods to reduce influence of noise and artifact in parameter maps
Biological interpretation and reporting	• *K*^Trans^ or *PS* reported as “leakage rate” of the GBCA • Full reporting of DCE-MRI and *T*_1_ measurement acquisition and analysis (Table 3)	• Data on precision and accuracy of *PS* including validation against other measures of BBB integrity • Reliable measurement of capillary surface area in vivo

Abbreviations: BBB, Blood-brain barrier; DCE-MRI, dynamic contrast-enhanced magnetic resonance imaging; GBCA, gadolinium-based contrast agent; *K*^Trans^, volume transfer constant; *PS*, permeability–surface area product; VIF, vascular input function.

### 3.1 MRI hardware

DCE-MRI measurements of subtle leakage are feasible at both 1.5 T and 3 T, and there is presently no published evidence indicating the benefit or otherwise of increased magnetic field strength. Although the influence of field strength on the signal-to-noise ratio is well understood, the effect on errors in the leakage rate is complex and mediated by field strength–dependent differences in the pre-contrast longitudinal relaxation rate *T*_1,0_, contrast agent relaxivity *r*_1_ [63], [64], transmit and receive coil inhomogeneity, and other factors; additional studies including numerical simulations of these effects would help to assess the influence of magnetic field strength. Because the signal changes measured are typically close to the noise level, future hardware developments that increase CNR while preserving stability and homogeneity will likely improve the precision of leakage measurements. It is plausible that imaging at 3 T and 7 T will, in some scenarios, yield increased CNR and greater precision of leakage parameters or, alternatively, facilitate scanning at higher spatial resolution with reduced partial volume artifact compared with 1.5-T imaging. Scanning at higher field also facilitates structural scanning at higher spatial resolution, which is likely to result in better evaluation of BBB changes in relation to cSVD features, including WMH and enlarged perivascular spaces. Temporal stability and artifact level are also crucial hardware considerations because of the very small signal enhancement and signal change observed. Use of high-sensitivity radiofrequency receive coils with a high number of elements and appropriate padding to restrict head motion is also likely to be beneficial. Finally, the capability to achieve a high maximum gradient strength and slew rate ensures short minimum echo times, which reduce confounding *T*_2_* effects of the GBCA.

### 3.2 Pulse sequences

A three-dimensional MRI pulse sequence is recommended to maximize the signal enhancement changes relative to the noise level, to reduce the effect of inflow artifact on the VIF and to obtain adequate spatial resolution and coverage within an acceptable scan time. Use of both three-dimensional sGRE (also known as “SPGR,” “FLASH,” and “T1-FFE”; e.g. [26]) and three-dimensional saturation-recovery sGRE (SR-sGRE; e.g. [12]) have been reported. Spoiled GRE with short echo time and repetition time permits faster imaging, but there is no evidence to indicate which of these sequences is more sensitive to BBB leakage. Furthermore, despite knowledge of the equations governing the signal intensity of these sequences as a function of relaxation times and acquisition parameters, determination of the optimal sequence and parameters for precise measurement of subtle leakage are not trivial and thus further theoretical and experimental exploration is required.

Although DCE-MRI typically consists of serial signal intensity measurements, from which *T*_1_ changes are calculated via the signal enhancement relative to baseline, some researchers have instead directly obtained a series of quantitative *T*_1_ measurements [65]. The latter approach may ameliorate some of the effects of scanner instability and coil inhomogeneity, as well as changes in *T*_2_* (since the signal is not normalized to the pre-contrast signal intensity), but *T*_1_ relaxation maps take significantly longer than *T*_1_w images to acquire. This approach may in principle be suited to long acquisitions when subjects are removed from the scanner between measurements or where the DCE-MRI scans can be interleaved with other (e.g., structural) MRI sequences. Dynamic acquisitions acquiring data continuously for longer than 15-20 minutes might increase detectability as more contrast enters the brain parenchyma or CSF; however, the benefits of extending the acquisition time will be limited by practical considerations such as cost and patient tolerability, and by renal clearance of GBCAs.

Finally, accurate quantification of *T*_1_ change and GBCA concentration requires knowledge of the pre-injection tissue and blood *T*_1_ [33] values with ideally the same spatial resolution and coverage as the dynamic acquisition; a minor drawback of this approach is the additional time and complexity needed to accurately and precisely measure *T*_1_ in both flowing and stationary tissues. Alternatively, literature *T*_1_ values may be used to reduce the examination time, but it should be noted that parenchymal *T*_1_ has a known association with cSVD burden and risk factors [26] and could therefore confound leakage measurements if incorrect, whereas blood *T*_1_ varies with age and hematocrit [66], [67]; assumed *T*_1_ values may therefore not be advisable in this context if reliable *T*_1_ measurements can be made. Variable flip angle sGRE and variable saturation-recovery delay SR-sGRE have been used to measure pre-contrast *T*_1_ [12], [17]. The sGRE technique is faster but known to be highly sensitive to flip angle inhomogeneity and inaccuracy [68]. Investigators should consider the impact of flip angle variation on both *T*_1_ and DCE-MRI measurements and, if necessary and feasible, perform a correction using a reliable flip angle mapping method [69], [70].

### 3.3 Acquisition parameters

DCE-MRI of the whole brain or, at minimum, the basal ganglia, periventricular tissues, and centrum semiovale from anterior to posterior regions is recommended for studies of cSVD. The spatial resolution required depends on the study aims but should be sufficient to resolve the smallest structures, tissues, or lesions of interest with minimal partial volume artifact. For all studies, the resolution and orientation should be such that at least one major blood vessel is clearly resolved for measurement of the VIF. Acquired voxel volumes of 2-10 mm^3^ are typical. For a three-dimensional acquisition, Gibbs artifact propagates in all three dimensions; therefore, thick slices should be avoided to reduce the impact on parameter maps [7].

An imaging volume with axial orientation typically permits faster sampling if inferior brain regions such as the posterior fossa are not of interest; however, slab-selective excitation of an axial imaging volume may result in blood inflow artifact in the VIF. This effect can be reduced via sagittal or coronal acquisition. The use of spatially nonselective excitation will further reduce inflow and may increase uniformity of the excitation profile.

Unfortunately, it is challenging to achieve the above spatial requirements with both a rapid sampling rate and acceptable CNR using currently available 1.5-T and 3-T MRI scanners. As a consequence, the rapid concentration changes following a bolus injection of GBCA are difficult to resolve. For subtle leakage measurement, the primary justification for high temporal resolution sampling is to measure the high and rapidly changing GBCA concentrations in blood during the first pass, so that leakage occurring during this period can be modeled. However, the error caused by temporal undersampling of the first pass can be assessed via simulations and it has been shown that this may be small with a temporal resolution on the order of one minute [7]. In an alternative approach, the first pass is sampled at higher temporal resolution with consequently reduced spatial coverage and/or spatial resolution during the early circulatory phases [30]. This approach increases the complexity of the acquisition, but provided the spatial and temporal resolutions are sufficient to accurately measure concentration in a large vessel, the contribution of leakage during the first pass can be appropriately modeled. A third option is to perform a slow injection of GBCA, which results in slower early-phase blood concentration changes; this approach allows the signal dynamics to be adequately sampled at lower temporal resolution, reduces *T*_2_* effects and the range of blood GBCA concentrations to be measured, and ensures that venous GBCA concentration more accurately reflects the arterial concentration and therefore the VIF. Although a slow injection approach has been reported previously in the literature [19], the benefits have yet to be evaluated for subtle BBB leakage measurement.

Although leakage rates can be measured at low temporal resolution, there are potential benefits to limiting the scan time for each volume: first, artifact and blurring due to patient motion and GBCA concentration change may be reduced; second, rapid sampling of both tissue and blood GBCA concentrations after a bolus injection permits measurement of CBF as well as plasma volume fraction and leakage rate, provided a reliable *arterial* (not venous) input function can be measured and an appropriate pharmacokinetic model is used [71].

A final important consideration is the total scan duration. For measurement of slow extravasation, a longer overall scan time was shown to increase the reproducibility of leakage measurements [36]; in practice, the acquisition time is limited by patient cooperation (e.g., head movement), the availability and cost of scanning time, and the need to obtain additional images for clinical evaluation and/or research purposes. As the leakage rate differs between GM, WM, and lesions, the optimal scan time may also depend on the clinical focus of the study. As a guide, we recommend that DCE-MRI scanning continues for at least 15 and preferably 20 minutes at 3 T [72]. Multiple images should also be acquired before a contrast injection to allow reliable estimation of the GBCA concentration, which is based on the relative signal enhancement with respect to pre-contrast images [35]. The requirement for a relatively long acquisition time may be one reason for the limited adoption of the DCE-MRI method in cSVD research studies, and future evaluation of ways to reduce the scan time would be beneficial. Interleaving of the DCE-MRI scan with other MRI techniques (e.g., FLAIR, *T*_2_w) to limit the total examination time, while still acquiring DCE-MRI images at late time points, is a potential solution but would require detailed consideration including an understanding of the effects of contrast agents on the interleaved sequences and the potential effect of any additional pre-scan adjustments. Long acquisition times might also be achieved more conveniently via methods that enable the patient to leave and re-enter the MRI scanner during the experiment.

In summary, there is considerable uncertainty around the “optimal” pulse sequence and acquisition parameters to use and further experimental and theoretical investigation is required. Cramer et al. and other groups used a Monte Carlo simulation approach to generate synthetic data for a range of pharmacokinetic parameters and incorporating various effects, such as CBF, noise, and scanner drift; these were then fitted using a pharmacokinetic model to yield graphs of “measured” versus “actual” leakage measures, to illustrate the precision (error bars) and accuracy (deviation from the line of equality) of the estimates [7], [34]. Barnes et al. also used a simulation approach, introducing the “K-CNR” quantity to represent the CNR of the measured leakage rate for a 10% difference in the actual leakage rate [35]; however, this approach combines both systematic and random errors in a single metric. All simulation approaches are limited by the accuracy of the model used to generate the ground truth data (typically the two-compartment exchange model) and rarely account for factors that may be substantial but difficult to predict and simulate, such as motion, ghosting, and Gibbs artifacts, and by the spatiotemporal noise structure.

### 3.4 Contrast agent

The leakage rate and the accuracy and precision of its measurement are likely to depend on the size, shape, and chemical properties of the contrast agent [73], [74], but at present, there is no convincing evidence for selecting a specific GBCA for studies of BBB integrity. On theoretical grounds, one would prefer a contrast agent with a strong *T*_1_ relaxivity, high BBB permeability (compared with the agents listed in Table 1), and long biological half-life to obtain the lowest detection limit for leakage. Binding of linear GBCAs to albumin is another issue that deserves consideration, as it will hinder extravasation on the one hand but will increase relaxivity due to slower molecular tumbling. Partial protein binding also adds uncertainty to the conversion between signal change and GBCA concentration, as the protein concentration may vary between compartments and tissues [75].

Because the aforementioned issues require further investigation, the choice of contrast agent should at present be based primarily on safety considerations, including minimizing the risks of nephrogenic systemic fibrosis [76] and long-term retention of gadolinium [77], [78], [79]. Some investigators have used a reduced dose of GBCA to reduce “ceiling” effects on the signal enhancement during the first pass [62], [65]. For measurement of slow leakage, however, increasing the gadolinium concentration gradient between the vascular space and parenchyma will drive increased transfer across the BBB and reduce the leakage detection limit; ceiling effects can be avoided by tailoring the pulse sequence parameters (e.g., flip angle for sGRE) during the first pass or by reducing the injection rate. Therefore, we recommend using the standard clinical dose of GBCA subject to patient safety considerations.

### 3.5 Data preprocessing

Once images have been acquired and before kinetic modeling of the data, a number of preprocessing steps (Fig. 3) should be performed as required. Head motion, which is inevitable over a lengthy DCE-MRI scan, should be corrected using widely available image co-registration algorithms such as SPM Realign (https://www.fil.ion.ucl.ac.uk/spm/) and FSL MCFLIRT [80]. The second preprocessing step is to convert the absolute MRI signal to the signal enhancement relative to the pre-injection intensity. Third, the signal enhancement is converted to tissue GBCA concentration. A GBCA induces a change in *R*_1_ (=1/*T*_1_) that is approximately linearly related to the concentration via a proportionality constant known as the relaxivity *r*_1_. Because there is little information available regarding the variation of *r*_1_ in different tissues, it is normally assumed to be the same in blood and brain tissue; therefore, the value used has no effect on the final leakage measurement. The *R*_1_ increase in turn causes a signal enhancement that is approximately linear at low GBCA concentrations but dependent on the pre-contrast *T*_1_. We recommend this conversion be performed using an equation that accurately describes the MR signal of the pulse sequence used. Measured pre-contrast *T*_1_ and, if required and available, flip angle values should be used in such calculations instead of assumed values, particularly for techniques with high *B*_1_ sensitivity such as sGRE. For blood, the determination of GBCA concentration from signal change can be more difficult as *T*_2_* and inflow effects may affect the relationship, especially at the relatively high first-pass concentrations. Measuring the phase of the MRI signal in large vessels provides a potential alternative way to determine the VIF, which has been proposed for DSC-MRI and DCE-MRI measurements and could in future be explored in this context [81], [82]. Enhancement to concentration conversion in blood can alternatively be achieved by scanning test objects containing a range of known gadolinium concentrations and with appropriate pre-contrast *T*_1_ values [36], but the concentration estimates are influenced by the accuracy of the assumed blood *T*_1_ as discussed previously.

### 3.6 Data analysis

Many different approaches have been reported for generating metrics of BBB integrity from DCE-MRI (Table 1) in cSVD and other pathologies, with the outcome measures variously labeled as “*K*^Trans^,” “permeability,” “leakage rate,” “BBB opening score,” “BBB permeability index,” etc. This variability impedes reliable interpretation, and comparison and pooling of data across studies. All such analysis methods can be categorized as either qualitative, semiquantitative, or quantitative. Because the tissue enhancement in cSVD is normally too small to be visible radiologically, qualitative analysis is rarely used except to identify visibly enhanced areas in CSF or stroke lesions.

Semiquantitative analysis has been used to probe BBB leakage in several cSVD studies, including area-under-curve calculation [22], mixed general linear modeling of the MRI signal to determine differences in the signal-time curves [23], and several other methods referred to in Table 1. Such approaches are relatively straightforward to implement and may reflect in part variation in BBB leakage across a sample over which the scan protocol is kept constant. However, the signal changes after GBCA injection depend strongly on the pulse sequence, field strength, contrast injection protocol, vascular supply, the time delay between contrast administration and measurement, and other factors additional to the kinetics of GBCA leakage; the signal changes and derived semiquantitative parameters are therefore not considered to be quantitative markers of BBB leakage.

Quantitative pharmacokinetic modeling approaches, which aim to generate (continuous) kinetic measures with a direct relationship to the underlying tissue properties [7], are simpler to interpret and less sensitive to the acquisition protocol than qualitative and semiquantitative analysis; this is particularly salient for comparison of results between sites and for longitudinal and multicentre studies where MRI system differences, scanner upgrades, and instrumental instability are likely to have a smaller effect on the values of carefully determined pharmacokinetic measures. The aim of such analysis is normally to estimate the BBB leakage rate by modeling the relationship between blood and tissue GBCA concentrations as a function of tissue and GBCA properties. In recent years, understanding and confidence in the use of pharmacokinetic modeling for subtle BBB leakage measurement has increased and its application has widened. We therefore recommend this approach where the imaging protocol is adequate to support it, for example, availability of a high-quality VIF, and with awareness of the limitations of this type of analysis discussed below.

### 3.7 Pharmacokinetic modeling

Several pharmacokinetic models are commonly used to analyze DCE-MRI images in a range of tissues and pathologies, and it is critical to select a model appropriate to the acquisition protocol, the tissue microstructure, and the likely ranges of the pharmacokinetic parameters [7], [8], [83]. Ideally, the model should predict the total tissue GBCA concentration *C*_t_ (i.e. including both capillaries and the EES) using the minimum number of parameters required to properly fit the data. For measurement of slow leakage at low temporal resolution, we recommend the Patlak model, which has now been used by several groups in the subtle BBB leakage literature and has been shown to perform well in comparison to other models, including the two-compartment exchange and extended Tofts models [7], [34], [35]. The Patlak model makes two particular assumptions: (1) GBCA concentration in capillaries is accurately represented by VIF measurements in a large vessel, which is justified where tissue perfusion is sufficiently high in relation to BBB leakage and to changes in arterial GBCA concentration, and (2) back-flux from the EES to the capillaries is negligible, which is normally justified in subtly leaking tissues (though not necessarily in tissues with higher leakage rates, as found in some stroke lesions [7]) where the plasma concentration remains much greater than that in the EES. These assumptions lead to a simple model equation that is conveniently linear in the two unknown parameters:

(1) $$C_{t}\left(t\right)=v_{p}c_{p}\left(t\right)+PS\int_{0}^{t}c_{p}\left(t^{'}\right)\text{d}t^{'}\text{,}$$

where the permeability–surface area product (*PS*) represents the BBB leakage rate per unit capillary plasma GBCA concentration and per unit tissue volume; *v*_p_ is the dimensionless parameter representing the capillary blood plasma volume fraction in tissue, and $c_{p}\left(t\right)$ is the GBCA concentration in blood plasma; the latter is given as $c_{p}\left(t\right)=c_{b}\left(t\right)/\left(1-\text{Hct}\right)$, where Hct is the hematocrit and $c_{b}\left(t\right)$ is the GBCA blood concentration estimated by the VIF. Example data and Patlak model fits are shown in Fig. 2.

It is essential to consider the appropriateness and limitations of this or any other pharmacokinetic model in relation to the particular tissue properties and acquisition protocol pertaining to the study. For example, the assumption of high tissue perfusion may be inappropriate for modeling the rapid concentration changes that occur around the time of the first pass after a bolus injection—this inaccuracy may be reduced by excluding the early data points from the fitting (however, the *c*_p_ values during the first pass are retained to calculate the integral term of Eq. 1) [7], [62]. *PS* measurements in highly ischemic tissues (i.e., those with very low CBF) could also be confounded. The further assumption of negligible back-flux across the BBB may also be invalid for the relatively high leakage rates sometimes found in stroke lesions and/or at long acquisition times where the GBCA concentrations in the EES and capillaries may be comparable; back-flux is expected to become significant as the acquisition time after injection approaches the mean transit time for the EES, equal to *v*_e_/*PS*, [8] and is likely to be (although there is no empirical data) much longer than the recommended 15- to 20-minute acquisition time in normal-appearing brain tissue.

To assess model suitability, we recommend that time-signal data and model fits to concentration-time curves be inspected visually in at least a subsample of the data; if required, simulations for testing model validity should be performed and reported (e.g., references [7], [32], [62]). Statistical approaches, such as the Akaike information criterion, may also aid model selection [7], [84], [85] but address only goodness of fit and not the model's physical or biological validity. Data can be fitted using widely available nonlinear least squares minimization algorithms, and convergence of the fit should be verified visually in a subset of the data. Constraints to fitted parameters should in general be avoided because noise and artifacts can result in values that lie outside of the expected ranges, for example, scanner drift can result in biased, potentially even negative, leakage rate and plasma volume fraction estimates [7], [35]. The Patlak model may be fitted with high computational efficiency using the “graphical” Patlak approach, in which *PS* is determined as the slope of a $C_{t}\left(t\right)/c_{p}\left(t\right)$ versus $\int_{0}^{t}c_{p}\left(t^{'}\right)dt^{'}/c_{p}\left(t\right)$ scatter plot. Alternatively, multiple linear regression analysis can be performed with $C_{t}\left(t\right)$ as the dependent variable and $c_{p}\left(t\right)$ and $\int_{0}^{t}c_{p}\left(t^{'}\right)\text{d}t^{'}$ as the regressors, which is less vulnerable to noise for small $c_{p}\left(t\right)$ than the graphical method.

Although the validity of the specific assumptions underlying the Patlak model can be assessed, several other assumptions and confounds can affect results from any model. For example, research is needed to determine the influence of water exchange rates across the endothelium and cell membrane on the estimated subtle leakage rates because water exchange is assumed to be infinitely rapid in most pharmacokinetic models [86]. Signal drift, which may result in comparable signal changes to those induced by any GBCA leakage, can also have a substantial influence on the estimated pharmacokinetic parameters [7], [33], [35], and research is needed to better understand the physiological and instrumental processes underlying drift and to develop methods for assessment and compensation. It is therefore prudent to perform simulations and “sham” DCE-MRI scans without contrast to evaluate the potential impact on leakage rate estimates. Other common assumptions, such as tissue-independent relaxivity, instantaneous mixing of the tracer within tissue compartments, and an equal hematocrit in large vessels and capillaries, may also affect accuracy. Pharmacokinetic models are necessarily based on a highly simplified description of tissue microstructure and function and therefore can ignore potentially relevant features such as the perivascular space and interstitial fluid transport.

### 3.8 Vascular input function

An important consideration for quantitative measurements is determination of the VIF because pharmacokinetic models require knowledge of the GBCA concentration in the blood plasma entering the tissue. Measurement in the feeding cerebral arteries is difficult because of their small cross-section leading to partial volume artifact and, particularly for axial acquisitions, the rapid inflow of protons from below the volume of excitation, which have not reached a steady state, giving rise to reduced enhancement after contrast administration and therefore inaccurate concentration estimates [71], [87]. We therefore recommend VIF measurement in a large venous sinus, such as the superior sagittal sinus because both of these detrimental effects can be minimized: partial volume artifact due to the increased diameter of the lumen and inflow artifact due to the lower blood velocity and longer time available to reach a steady state within the excitation volume, provided this is sufficiently large. The posterior section of the superior sagittal sinus is also less sensitive to motion because of its position near the fulcrum of head movement when the subject is lying supine. In our experience, venous VIFs have higher signal-to-noise ratio and CNR and are more representative of blood concentration than VIFs measured in the internal carotid or middle cerebral arteries, for the reasons described previously. Although venous GBCA concentration theoretically represents an “output” rather than an input function, the arterial and venous concentration profiles are found to be very similar following the rapid early changes during the first pass of the bolus [71]. For the purposes of applying the Patlak model, which should not in any case be used to fit first-pass data, the superior sagittal sinus VIF provides a good approximation to the arterial input function. VIF voxels should be selected using the dynamic rather than structural images to ensure good CNR and to avoid contamination from non-blood signals.

A VIF may potentially be estimated using a mathematical function based on population-averaged data [88], [89], [90], and this approach avoids some of the errors inherent in patient-specific VIF measurements. However, assumed VIF functions do not take account of either day-to-day or intersubject differences [91] because of variation in cardiac and renal function, body composition, and other unknown factors. Such confounds could potentially influence cross-sectional and longitudinal analysis of BBB leakage; therefore, we recommend measurement of a high-quality VIF in each patient.

### 3.9 Regional measurement

**Fig. 4:**
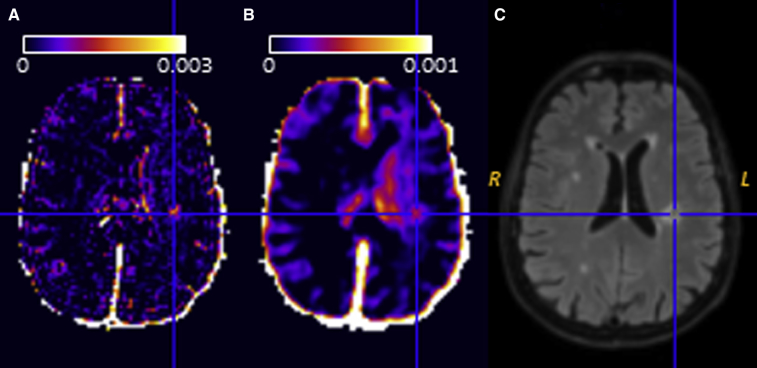
(A) Illustrative 3-T *PS* (units min^−1^) map in a patient with cSVD (71-year-old female) after acute lacunar stroke 6 weeks previously. For the corresponding *PS* map (B), the raw DCE-MRI images were smoothed using a three-dimensional gaussian kernel (full width at half-maximum 2 mm) during preprocessing to suppress the noise and Gibbs artifact apparent in (A). In both maps, the low level of leakage is apparent, with noticeably higher values in the stroke lesion (indicated by the cross hairs) and in the periventricular normal-appearing white matter ipsilateral to the stroke lesion. The corresponding *T*_2_w-FLAIR image is shown in (C). DCE-MRI data were acquired and processed by the authors as described in the caption to Fig. 2B. Abbreviations: cSVD, cerebral small vessel disease; DCE-MRI, dynamic contrast-enhanced magnetic resonance imaging; *PS*, permeability–surface area product.

Two general approaches to image analysis are commonly used in DCE-MRI: (1) voxel-based mapping of pharmacokinetic parameters, which are then sampled using tissue masks or regions of interest and (2) modeling of ROI- or tissue-averaged signals. The first approach is, in principle, superior because it does not assume that voxels within a tissue or region share a common set of properties, the spatial resolution of the original images is retained, additional insight into the spatial pattern of leakage may be obtained, and histogram analyses of leakage parameters can be performed [12], [25]. In practice, as illustrated in Fig. 4, the noise and artifact levels for individual voxels can be substantial and may impede the generation of reliable parameter maps; in such cases, signal averaging to increase the CNR within regions before pharmacokinetic modeling (as shown in Fig. 2) is advisable and, by reducing the size of the data set, renders visual inspection of the fit quality feasible. Mean or median *PS* and *v*_P_ and a measure of inter-subject variation (such as standard deviation or interquartile range) should be reported for each region or tissue as a minimum.

Where voxel-based analysis is performed, the low CNR leads to leakage estimates that are frequently close to the noise level and may often be negative. Van de Haar et al. [12], [30] illustrated the substantial noise contribution in individual voxels resulting in negative leakage estimates in a relatively large proportion. Using a histogram approach, the authors attributed negative leakage values plus a mirrored positive distribution of these voxels to noise, whereas the remaining positive values in the histogram were classified as having detectable leakage; the leakage values of these “noise” voxels were set to zero before calculation of the mean leakage rates over remaining voxels and, additionally, the fraction of “detectably leaking” voxels for each region was calculated. Raja et al. agreed that noise and negative values should be addressed but favored a different statistical procedure whereby the Akaike information criterion is used to determine whether the data in individual voxels support a model (e.g., Patlak) that includes BBB leakage versus a vascular-only model with no leakage term (i.e., *PS* = 0), although it is unclear how this addresses the problem of a model that fits the data well but with negative *PS* [16]. Another approach was proposed by Taheri et al., who fitted a statistical distribution to *PS* values measured in a sample of healthy volunteers, and used the 95% confidence limit of this distribution to identify voxels with “abnormal” leakage [65]. Each of these methods has limitations leading to arbitrary classification of “leaking” versus “nonleaking” or “noise” voxels that depend on instrumental and other site- and study-specific factors including the artifact level, CNR, the complicated noise structure, and the characteristics of “healthy” reference subjects, with the potential to bias study outcomes. The validity of such methods in the presence of significant signal drift, where *PS* measurements are likely to be offset from the true values, is also unclear. However, for research where the outcome measures relate to the properties of regions and tissues, it is not essential to dichotomize voxels in this manner, and several studies have simply averaged signals or leakage rates across all voxels within the regions and tissues of interest to reduce the effect of noise. As there is presently no consensus on voxel classification methods, a simple averaging approach is recommended as a common “baseline” for future studies.

The definition of region and tissue masks will depend primarily on the research questions and study objectives. Where tissue masks are used, care should be taken to avoid contamination by adjacent structures (e.g., CSF and major blood vessels), for example, by “eroding” the mask images. The use of carefully placed small regions of interest is an alternative approach that permits investigation of smaller structures and regions while avoiding contamination but is more observer dependent than automated methods and suffers from higher noise levels.

### 3.10 Biological interpretation

Analysis of DCE-MRI using an appropriate pharmacokinetic model yields estimates of the BBB leakage rate, equivalent to the *PS* of the regional microvasculature. The permeability *P* is defined as the quantity of GBCA molecules leaving the capillary through the BBB per unit time, per unit capillary wall area, and per unit capillary blood plasma concentration of the GBCA, and the surface area *S* is the total capillary wall surface area per unit tissue volume; the product *PS* therefore represents the overall normalized “leakage rate” of GBCA across the BBB, i.e., the quantity leaking per unit time, per unit capillary plasma GBCA concentration, and per unit tissue volume. Because *S* is unknown, it is impossible to calculate the permeability *P* or to distinguish between differences or changes in the permeability and differences or changes in the capillary wall surface area. Currently, it is not possible to determine the local capillary wall surface area of the brain microvasculature in vivo with confidence; MR techniques for estimating the related vessel size have been proposed [92] but are not straightforward to implement, involve other assumptions, and are at an early stage of development. “Leakage” and “leakage rate” of GBCA are therefore more appropriate terminology than “permeability” for describing DCE-MRI measurements of *PS*. It should also be emphasized that the leakage rate of a particular GBCA is being measured—typically, GBCAs are relatively small gadolinium complexes with low molecular weight (e.g., the molecular weight of gadobutrol and gadoteric acid are 604.7 g mol^−1^ and 558.6 g mol^−1^, respectively). Thus, although *PS* is a quantitative measure that is influenced by BBB integrity, it cannot predict the BBB transport rates of specific biological molecules in the blood such as glucose and proteins.

### 3.11 Reporting

Reporting of the employed methodology to measure subtle BBB leakage has sometimes been unclear and inadequate to permit replication. Although general reporting standards for clinical studies are well known, we recommend in particular that full details of the MRI acquisition, contrast administration, image processing, and pharmacokinetic modeling should be reported in sufficient detail to enable other groups to repeat the experiments. Reporting should include full details of the dynamic and *T*_1_ measurement scans, image preprocessing, VIF selection, model fitting, formulas, region and tissue mask generation, and postprocessing and statistical analysis of the data. In particular, the way in which the leakage value is calculated and to which voxels and tissue regions it refers requires attention. Where possible, individual patient hematocrits should be measured and used to report *PS* [7]. Some publications have used the alternative *K*^Trans^ (defined as the quantity of GBCA leaking across the BBB per unit time, tissue volume, and *arterial* blood plasma concentration) notation, which is interchangeable with *PS* provided the model assumptions discussed previously (e.g., high blood flow, slow leakage) are applicable; in this case, we recommend reporting *PS* as a parameter that has a clear biological interpretation. This measure should be reported in preference to *K*_i_, which quantifies leakage in relation to blood concentration rather than blood plasma concentration of GBCA (because intravascular GBCA is normally restricted to the plasma compartment). Previous reviews provide guidance on appropriate units for reporting such data [8], [93]. We recommend to report representative values (i.e., mean or median) and spread (e.g., standard deviation or interquartile range) of all fitted pharmacokinetic parameters, including *v*_p_ or the related blood volume fraction $v_{b}=\;v_{p}/\left(1-\text{Hct}\right)$. The tissue regions to which these values pertain should be clearly described; where parameter maps are shown, thresholds and other methods or filters used to generate these should be noted. We recommend that the acquisition parameters and other information summarized in Table 3 should be reported with the study findings.

**Table 3:** Table of key parameters and information (not exhaustive) that should be reported where applicable

MRI acquisition	• Pulse sequence used for DCE-MRI, and *T*_1_ and flip angle mapping • Field strength, inversion/saturation-recovery delay, repetition time, echo time, flip angle • *k*-space sampling scheme • Acceleration techniques, bandwidth • Orientation, acquisition matrix, field of view, *acquired* spatial resolution (including structural scans) • Temporal resolution, acquisition time • Signal drift
Contrast agent	• Agent, concentration, dose, volume • Injection rate, time, and delay • Number of pre-contrast images
Preprocessing	• Algorithms and formulas used for realignment, *T*_1_ calculation, concentration estimation etc.
Pharmacokinetic modeling	• Model selection and justification • Fitting method, data points excluded, constraints • Details of simulations performed
Vascular input function	• Location, size, and procedure for selecting
Regional measurement	• Procedure for generating ROIs and tissue masks • Specify the signal modeled, i.e., voxel signal or region-averaged signal
Results	• Summary *PS* or *K*^Trans^, *v*_P_, *v*_b_, *T*_1_, and other relevant parameter values for each region • Representative signal enhancement curves including VIF • Representative concentration curves with model fit

Abbreviations: DCE-MRI, dynamic contrast-enhanced magnetic resonance imaging; *K*^Trans^, volume transfer constant; *PS*, permeability–surface area product; VIF, vascular input function.

## 4 Practical considerations for DCE-MRI in cognitively impaired and demented subjects

DCE-MRI has been applied in several cognitive impairment and dementia patient groups (Table 1), including mild cognitive impairment, vascular cognitive impairment, and early Alzheimer's disease [11], [12], [13], [14], [15], [17], [18], [19], [20], [29], [30]. However, researchers should be aware that there are some practical difficulties of studying and imaging such patients; in particular, detection of very small signal changes over a long acquisition requires a degree of compliance, as DCE-MRI is sensitive to patient motion. Anecdotally, we find that regular communication with imaging staff during the examination, accompaniment before MRI by a familiar clinician or carer, and the opportunity to rest, move, or break between scans have a positive effect on tolerability. Padding to reduce head motion is essential, and real-time motion correction (e.g. navigator-based or optical) scanning methods may in future increase feasibility in less compliant subjects.

Image analysis steps including spatial normalization and tissue segmentation also pose specific challenges in patients with severe neurodegeneration because of variable and often substantial levels of brain atrophy and lesion burden.

Regarding implementation, literature in the field is confined to a relatively small number of research centers with relevant medical physics and image processing experience. However, specialist MRI hardware and pulse sequences are not required for implementation, and we believe there is now sufficient information and advice available in the literature, including in the present article, to enable most groups with a good level of general imaging expertise to employ the technique. To support this, MRI protocols and analysis tools for structural and quantitative imaging, including DCE-MRI, are published on the HARNESS website (www.harness-neuroimaging.org).

## 5 Priorities for future research

O'Connor et al. described a detailed roadmap for discovery, validation, and qualification of reliable IBs for use in cancer research and health care [10], which we believe provides a useful framework for development of IBs in other fields including cSVD and dementia. In the language of the roadmap, our review describes an IB that has passed through the initial “discovery” domain and now sits within the second “validation” domain, wherein three tracks (technical validation, biological and clinical validation, and cost effectiveness) are identified. As discussed in detail in the preceding sections and summarized below, progress has been made in some aspects of the validation domain, but we believe that further research is required for DCE-MRI measurement of subtle BBB leakage to cross the first “translational gap” whereby it can be recognized as a reliable measure for testing hypotheses in clinical research.

With regard to **technical validation**, there is presently only one published study of precision in vivo [36], studies of bias have been conducted mainly from a theoretical standpoint and are limited in scope; however, reasonable availability of the technique in the research setting is evidenced by publications from several groups globally. **Biological and clinical validation** has received some attention in the literature, with several studies showing associations between BBB integrity measures and certain clinical variables, but the diversity of acquisition and analysis protocols, and of study design, hampers comparison and pooling of such data; evidence linking IB measurements to the underlying biology is more sparse because of the lack of adequate reference methods. The **cost effectiveness** track has also received little attention to date: it is generally acknowledged that the long acquisition increases costs and discourages more widespread use, but a lack of data on precision is an obstacle to reliable calculation of statistical power and study cost. Cost-effectiveness will become more relevant after the first translational gap has been crossed and use of the IB in health care systems is considered.

As recommended by O'Connor et al., the three tracks should be pursued in parallel, but in this context especially they are interdependent and require a foundation of methodological research. Throughout this review and in Table 2, we have identified specific areas requiring further research. In the following paragraphs, we propose three areas of immediate priority for future work in the field:

1. A **standardized multivendor protocol** would underpin future studies of precision, bias, and clinical and biological validity and facilitate wider adoption. However, despite increasing application of the technique in clinical studies, little method development work is reported in the literature, leading to a lack of objective evidence on which to base a “consensus protocol.” In this review, we provide several specific consensus recommendations for acquisition and data analysis (Table 2) with the aim of encouraging greater harmonization, but there is insufficient evidence to recommend a specific protocol at present. We therefore recommend that an international working group be established to continue to work toward an open-access consensus protocol. The group should be open to all experts in the field, and recommendations should be dynamic and version-controlled to represent the current best standard and respond to emerging evidence including from current projects by North American (https://markvcid.partners.org), European (https://www.svds-at-target.eu/), and global (http://www.small-vessel-disease.org) consortia.
2. Evidence of **repeatability and reproducibility** should be obtained in healthy volunteers and/or patients. The outcome variables identified in Section 3.11 should be analyzed, and the raw images ideally made accessible to the research community to enable testing of different analysis methods and software.
3. Further evidence of **accuracy** is required, including research into bias and biological validity. Such evidence would increase confidence within the cSVD and dementia research community that measurements are not only feasible and reproducible but reflect the pathophysiological aspect of interest. Such investigations should include theoretical studies, comparison with independent markers of BBB dysfunction, development and use of suitable MRI test objects, and preclinical histological validation. Further clinical studies of BBB leakage (following the recommendations provided) in relation to disease severity, disease progression and other clinical variables would also increase confidence in the technique.

Although the aforementioned steps are essential to establish DCE-MRI subtle leakage measurement as an IB for research, we also encourage basic research to advance or even replace current techniques, in order to increase the precision, accuracy, and feasibility of imaging BBB dysfunction.

## 6 Conclusions

An increasing number of clinical studies are being published that indicate the possibility of, and growing interest in, MRI quantification of subtle BBB dysfunction for research into the pathophysiology of cSVD and dementia and, ultimately, for development and monitoring of treatments. As reported in this review, DCE-MRI provides at present the most promising means for achieving this and has yielded a number of intriguing findings in relation to cSVD. However, the technique has been implemented with diverse acquisition and analysis methods, which involve many assumptions and suffer from significant, sometimes unquantifiable, limitations that can render clinical findings difficult to interpret and impede comparisons between studies and centers. Some of the limitations stem from the practical and technical challenges of measuring very subtle leakage including the unfavorable CNR and artifact characteristics of the acquired signals. Reported BBB leakage measurements have a high variance between research sites, as noted by Raja et al. in their recent systematic literature review of BBB function in dementia [16]. Their article emphasized the predominant role of DCE-MRI with pre-contrast *T*_1_ quantification and pharmacokinetic modeling but further noted that reliable measurement of subtle leakage is a particular challenge with this method; the authors also emphasized the need for collaborative efforts to harmonize data collection and analysis methods, a task we have initiated here.

As a group with substantial collective technical and clinical experience in the cSVD and MRI fields, our intention in writing this review is to provide researchers with comprehensive information, advice, and consensus-based recommendations for performing such measurements in research studies, to describe the limitations so that authors and readers may better assess the quality and implications of studies, and to identify areas where further research and development will benefit future clinical applications. For clarity, we note that our recommendations are not intended as a medical guideline and that DCE-MRI quantification of subtle BBB leakage is not yet suitable for use as a clinical decision-making tool. We hope that these recommendations will encourage a greater degree of harmonization in future studies where possible, in order that data from multiple centers can be more easily compared and pooled. We have focused on DCE-MRI as the method that is, at present, most advanced and most widely used, and which we believe provides a quantitative, though relative, measure of BBB integrity. Nevertheless, the technique is relatively immature in the context of measuring subtle BBB leakage, and we note that our recommendations do not represent the final word on the subject but rather a pragmatic “baseline” approach that may inform the design of future studies, lead to greater harmonization and interstudy comparability, and provide a starting point for future initiatives to further standardize, develop, and validate the method. The alternative techniques for assessing BBB integrity described in this article may also undergo further development and merit greater prominence in future reviews.

**Research in Context**

1. Systematic review: This article summarizes the work performed by an international multidisciplinary working party that convened on four occasions, comprising a review of the literature and proposal of recommendations concerning future application and development of blood-brain barrier integrity imaging.
2. Interpretation: Examination of the literature revealed a high degree of methodological heterogeneity with potential to affect the findings and conclusions of research studies. Detailed advice and consensus recommendations are proposed to increase the quality and harmonisation of future clinical research studies.
3. Future directions: Areas are identified where insufficient evidence precludes firm recommendations and further research is required. Three priorities for further development towards a reliable imaging biomarker of subtle blood-brain barrier failure are identified.

## References

[1] Wardlaw J.M., Smith C., Dichgans M. (2013). Mechanisms of sporadic cerebral small vessel disease: insights from neuroimaging. Lancet Neurol.

[2] Wardlaw J.M., Smith E.E., Biessels G.J., Cordonnier C., Fazekas F., Frayne R. (2013). Neuroimaging standards for research into small vessel disease and its contribution to ageing and neurodegeneration. Lancet Neurol.

[3] Blair G.W., Hernandez M.V., Thrippleton M.J., Doubal F.N., Wardlaw J.M. (2017). Advanced neuroimaging of cerebral small vessel disease. Curr Treat Options Cardiovasc Med.

[4] Blair G.W., Appleton J.P., Law Z.K., Doubal F., Flaherty K., Dooley R. (2018). Preventing cognitive decline and dementia from cerebral small vessel disease: The LACI-1 Trial. Protocol and statistical analysis plan of a phase IIa dose escalation trial testing tolerability, safety and effect on intermediary endpoints of isosorbide mononitrate and cilostazol, separately and in combination. Int J Stroke.

[5] Farrall A.J., Wardlaw J.M. (2009). Blood-brain barrier: Ageing and microvascular disease–systematic review and meta-analysis. Neurobiol Aging.

[6] Heye A.K., Culling R.D., Valdes Hernandez Mdel C., Thrippleton M.J., Wardlaw J.M. (2014). Assessment of blood-brain barrier disruption using dynamic contrast-enhanced MRI. A systematic review. Neuroimage Clin.

[7] Heye A.K., Thrippleton M.J., Armitage P.A., Valdes Hernandez M.D.C., Makin S.D., Glatz A. (2016). Tracer kinetic modelling for DCE-MRI quantification of subtle blood-brain barrier permeability. Neuroimage.

[8] Sourbron S.P., Buckley D.L. (2013). Classic models for dynamic contrast-enhanced MRI. NMR Biomed.

[9] Smith E.E., Biessels G.J., De Guio F., de Leeuw F.E., Duchesne S., During M. (2019). Harmonizing brain magnetic resonance imaging methods for vascular contributions to neurodegeneration. Alzheimers Dement (Amst).

[10] O'Connor J.P., Aboagye E.O., Adams J.E., Aerts H.J., Barrington S.F., Beer A.J. (2017). Imaging biomarker roadmap for cancer studies. Nat Rev Clin Oncol.

[11] Taheri S., Gasparovic C., Huisa B.N., Adair J.C., Edmonds E., Prestopnik J. (2011). Blood-brain barrier permeability abnormalities in vascular cognitive impairment. Stroke.

[12] Zhang C.E., Wong S.M., van de Haar H.J., Staals J., Jansen J.F., Jeukens C.R. (2017). Blood-brain barrier leakage is more widespread in patients with cerebral small vessel disease. Neurology.

[13] Huisa B.N., Caprihan A., Thompson J., Prestopnik J., Qualls C.R., Rosenberg G.A. (2015). Long-term blood-brain barrier permeability changes in Binswanger disease. Stroke.

[14] Hanyu H., Asano T., Tanaka Y., Iwamoto T., Takasaki M., Abe K. (2002). Increased blood-brain barrier permeability in white matter lesions of Binswanger's disease evaluated by contrast-enhanced MRI. Dement Geriatr Cogn Disord.

[15] Starr J.M., Farrall A.J., Armitage P., McGurn B., Wardlaw J. (2009). Blood-brain barrier permeability in Alzheimer's disease: A case-control MRI study. Psychiatry Res.

[16] Raja R., Rosenberg G.A., Caprihan A. (2017). MRI measurements of Blood-Brain Barrier function in dementia: A review of recent studies. Neuropharmacology.

[17] Montagne A., Barnes S.R., Sweeney M.D., Halliday M.R., Sagare A.P., Zhao Z. (2015). Blood-brain barrier breakdown in the aging human hippocampus. Neuron.

[18] Zhang C.E., Wong S.M., Uiterwijk R., Backes W.H., Jansen J.F.A., Jeukens C. (2019). Blood-brain barrier leakage in relation to white matter hyperintensity volume and cognition in small vessel disease and normal aging. Brain Imaging Behav.

[19] Wang H., Golob E.J., Su M.Y. (2006). Vascular volume and blood-brain barrier permeability measured by dynamic contrast enhanced MRI in hippocampus and cerebellum of patients with MCI and normal controls. J Magn Reson Imaging.

[20] Bronge L., Wahlund L.O. (2000). White matter lesions in dementia: an MRI study on blood-brain barrier dysfunction. Dement Geriatr Cogn Disord.

[21] Munoz Maniega S., Chappell F.M., Valdes Hernandez M.C., Armitage P.A., Makin S.D., Heye A.K. (2017). Integrity of normal-appearing white matter: Influence of age, visible lesion burden and hypertension in patients with small-vessel disease. J Cereb Blood Flow Metab.

[22] Topakian R., Barrick T.R., Howe F.A., Markus H.S. (2010). Blood-brain barrier permeability is increased in normal-appearing white matter in patients with lacunar stroke and leucoaraiosis. J Neurol Neurosur Psychiatry.

[23] Wardlaw J.M., Doubal F., Armitage P., Chappell F., Carpenter T., Munoz Maniega S. (2009). Lacunar stroke is associated with diffuse blood-brain barrier dysfunction. Ann Neurol.

[24] Wardlaw J.M., Doubal F.N., Valdes-Hernandez M., Wang X., Chappell F.M., Shuler K. (2013). Blood-brain barrier permeability and long-term clinical and imaging outcomes in cerebral small vessel disease. Stroke.

[25] Wardlaw J.M., Farrall A., Armitage P.A., Carpenter T., Chappell F., Doubal F. (2008). Changes in background blood-brain barrier integrity between lacunar and cortical ischemic stroke subtypes. Stroke.

[26] Wardlaw J.M., Makin S.J., Hernandez M.C.V., Armitage P.A., Heye A.K., Chappell F.M. (2017). Blood-brain barrier failure as a core mechanism in cerebral small vessel disease and dementia: Evidence from a cohort study. Alzheimers Dement.

[27] Starr J.M., Wardlaw J., Ferguson K., MacLullich A., Deary I.J., Marshall I. (2003). Increased blood-brain barrier permeability in type II diabetes demonstrated by gadolinium magnetic resonance imaging. J Neurol Neurosurg Psychiatry.

[28] Israeli D., Tanne D., Daniels D., Last D., Shneor R., Guez D. (2011). The application of MRI for depiction of subtle blood brain barrier disruption in stroke. Int J Biol Sci.

[29] van de Haar H.J., Jansen J.F.A., van Osch M.J.P., van Buchem M.A., Muller M., Wong S.M. (2016). Neurovascular unit impairment in early Alzheimer's disease measured with magnetic resonance imaging. Neurobiol Aging.

[30] van de Haar H.J., Burgmans S., Jansen J.F., van Osch M.J., van Buchem M.A., Muller M. (2016). Blood-brain barrier leakage in patients with early alzheimer disease. Radiology.

[31] Li Y., Li M., Zuo L., Shi Q., Qin W., Yang L. (2018). Compromised blood-brain barrier integrity is associated with total magnetic resonance imaging burden of cerebral small vessel disease. Front Neurol.

[32] Staals J., Makin S.D., Doubal F.N., Dennis M.S., Wardlaw J.M. (2014). Stroke subtype, vascular risk factors, and total MRI brain small-vessel disease burden. Neurology.

[33] Armitage P.A., Farrall A.J., Carpenter T.K., Doubal F.N., Wardlaw J.M. (2011). Use of dynamic contrast-enhanced MRI to measure subtle blood-brain barrier abnormalities. Magn Reson Imaging.

[34] Cramer S.P., Larsson H.B. (2014). Accurate determination of blood-brain barrier permeability using dynamic contrast-enhanced T1-weighted MRI: A simulation and in vivo study on healthy subjects and multiple sclerosis patients. J Cereb Blood Flow Metab.

[35] Barnes S.R., Ng T.S., Montagne A., Law M., Zlokovic B.V., Jacobs R.E. (2016). Optimal acquisition and modeling parameters for accurate assessment of low Ktrans blood-brain barrier permeability using dynamic contrast-enhanced MRI. Magn Reson Med.

[36] Wong S.M., Jansen J.F.A., Zhang C.E., Staals J., Hofman P.A.M., van Oostenbrugge R.J. (2017). Measuring subtle leakage of the blood-brain barrier in cerebrovascular disease with DCE-MRI: Test-retest reproducibility and its influencing factors. J Magn Reson Imaging.

[37] Lupo J.M., Cha S., Chang S.M., Nelson S.J. (2005). Dynamic susceptibility-weighted perfusion imaging of high-grade gliomas: Characterization of spatial heterogeneity. AJNR Am J Neuroradiol.

[38] Thornhill R.E., Chen S., Rammo W., Mikulis D.J., Kassner A. (2010). Contrast-enhanced MR imaging in acute ischemic stroke: T2* measures of blood-brain barrier permeability and their relationship to T1 estimates and hemorrhagic transformation. AJNR Am J Neuroradiol.

[39] Arba F., Leigh R., Inzitari D., Warach S.J., Luby M., Lees K.R. (2017). Blood-brain barrier leakage increases with small vessel disease in acute ischemic stroke. Neurology.

[40] Rost N.S., Cougo P., Lorenzano S., Li H., Cloonan L., Bouts M.J. (2018). Diffuse microvascular dysfunction and loss of white matter integrity predict poor outcomes in patients with acute ischemic stroke. J Cereb Blood Flow Metab.

[41] Quarles C.C., Gochberg D.F., Gore J.C., Yankeelov T.E. (2009). A theoretical framework to model DSC-MRI data acquired in the presence of contrast agent extravasation. Phys Med Biol.

[42] Sourbron S., Heilmann M., Biffar A., Walczak C., Vautier J., Volk A. (2009). Bolus-tracking MRI with a simultaneous T1- and T2*-measurement. Magn Reson Med.

[43] Hales P.W., Clark C.A. (2013). Combined arterial spin labeling and diffusion-weighted imaging for noninvasive estimation of capillary volume fraction and permeability-surface product in the human brain. J Cerebr Blood F Met.

[44] Lawrence K.S., Owen D., Wang D.J.J. (2012). A two-stage approach for measuring vascular water exchange and arterial transit time by diffusion-weighted perfusion MRI. Magn Reson Med.

[45] Wang J.J., Fernandez-Seara M.A., Wang S.M., St Lawrence K.S. (2007). When perfusion meets diffusion: in vivo measurement of water permeability in human brain. J Cerebr Blood F Met.

[46] Gregori J., Schuff N., Kern R., Gunther M. (2013). T2-based arterial spin labeling measurements of blood to tissue water transfer in human brain. J Magn Reson Imaging.

[47] Liu P., Uh J., Lu H. (2011). Determination of spin compartment in arterial spin labeling MRI. Magn Reson Med.

[48] Schmid S., Teeuwisse W.M., Lu H., van Osch M.J. (2015). Time-efficient determination of spin compartments by time-encoded pCASL T2-relaxation-under-spin-tagging and its application in hemodynamic characterization of the cerebral border zones. Neuroimage.

[49] Lin Z., Li Y., Su P., Mao D., Wei Z., Pillai J.J. (2018). Non-contrast MR imaging of blood-brain barrier permeability to water. Magn Reson Med.

[50] Kim Y.R., Tejima E., Huang S., Atochin D.N., Dai G., Lo E.H. (2008). In vivo quantification of transvascular water exchange during the acute phase of permanent stroke. Magn Reson Med.

[51] Freeze W.M., Schnerr R.S., Palm W.M., Jansen J.F., Jacobs H.I., Hoff E.I. (2017). Pericortical enhancement on delayed postgadolinium fluid-attenuated inversion recovery images in normal aging, mild cognitive impairment, and Alzheimer disease. AJNR Am J Neuroradiol.

[52] Cho A.H., Cho Y.P., Lee D.H., Kwon T.W., Kwon S.U., Suh D.C. (2014). Reperfusion injury on magnetic resonance imaging after carotid revascularization. Stroke.

[53] Latour L.L., Kang D.W., Ezzeddine M.A., Chalela J.A., Warach S. (2004). Early blood-brain barrier disruption in human focal brain ischemia. Ann Neurol.

[54] Iannotti F., Fieschi C., Alfano B., Picozzi P., Mansi L., Pozzilli C. (1987). Simplified, noninvasive pet measurement of blood-brain-barrier permeability. J Comput Assist Tomo.

[55] Pozzilli C., Picozzi P. (1987). Modifications of blood-brain-barrier in multiple-sclerosis - a study with positron emission tomography. Med-Riv Enc Med Ital.

[56] Schlageter N.L., Carson R.E., Rapoport S.I. (1987). Examination of blood-brain-barrier permeability in dementia of the Alzheimer Type with [Ga-68] Edta and positron emission tomography. J Cerebr Blood F Met.

[57] Zhou Y., Huang S.C., Hoh C.K., Cloughesy T., Yang J., Phelps M.E. (1996). Gallium-68 EDTA PET study of brain tumor BBB permeability changes induced by intra-arterial RMP7. J Nucl Med.

[58] Partridge W.M. (2006). Introduction to the Blood-Brain Barrier: Methodology, Biology and Pathology.

[59] Dysken M.W., Nelson M.J., Hoover K.M., Kuskowski M., McGeachie R. (1990). Rapid dynamic CT scanning in primary degenerative dementia and age-matched controls. Biol Psychiatry.

[60] Caserta M.T., Caccioppo D., Lapin G.D., Ragin A., Groothuis D.R. (1998). Blood-brain barrier integrity in Alzheimer's disease patients and elderly control subjects. J Neuropsychiatry Clin Neurosci.

[61] van de Haar H.J., Burgmans S., Hofman P.A.M., Verhey F.R.J., Jansen J.F.A., Backes W.H. (2015). Blood-brain barrier impairment in dementia: Current and future in vivo assessments. Neurosci Biobehav R.

[62] Larsson H.B., Courivaud F., Rostrup E., Hansen A.E. (2009). Measurement of brain perfusion, blood volume, and blood-brain barrier permeability, using dynamic contrast-enhanced T(1)-weighted MRI at 3 tesla. Magn Reson Med.

[63] Rohrer M., Bauer H., Mintorovitch J., Requardt M., Weinmann H.J. (2005). Comparison of magnetic properties of MRI contrast media solutions at different magnetic field strengths. Invest Radiol.

[64] Pintaske J., Martirosian P., Graf H., Erb G., Lodemann K.P., Claussen C.D. (2006). Relaxivity of Gadopentetate Dimeglumine (Magnevist), Gadobutrol (Gadovist), and Gadobenate Dimeglumine (MultiHance) in human blood plasma at 0.2, 1.5, and 3 Tesla. Invest Radiol.

[65] Taheri S., Gasparovic C., Shah N.J., Rosenberg G.A. (2011). Quantitative measurement of blood-brain barrier permeability in human using dynamic contrast-enhanced MRI with fast T1 mapping. Magn Reson Med.

[66] Lu H., Clingman C., Golay X., van Zijl P.C. (2004). Determining the longitudinal relaxation time (T1) of blood at 3.0 Tesla. Magn Reson Med.

[67] Wu W.C., Jain V., Li C., Giannetta M., Hurt H., Wehrli F.W. (2010). In vivo venous blood T1 measurement using inversion recovery true-FISP in children and adults. Magn Reson Med.

[68] Stikov N., Boudreau M., Levesque I.R., Tardif C.L., Barral J.K., Pike G.B. (2015). On the accuracy of T1 mapping: searching for common ground. Magn Reson Med.

[69] Deoni S.C. (2007). High-resolution T1 mapping of the brain at 3T with driven equilibrium single pulse observation of T1 with high-speed incorporation of RF field inhomogeneities (DESPOT1-HIFI). J Magn Reson Imaging.

[70] Yarnykh V.L. (2007). Actual flip-angle imaging in the pulsed steady state: A method for rapid three-dimensional mapping of the transmitted radiofrequency field. Magn Reson Med.

[71] Sourbron S., Ingrisch M., Siefert A., Reiser M., Herrmann K. (2009). Quantification of cerebral blood flow, cerebral blood volume, and blood-brain-barrier leakage with DCE-MRI. Magn Reson Med.

[72] van de Haar H.J., Jansen J.F.A., Jeukens C., Burgmans S., van Buchem M.A., Muller M. (2017). Subtle blood-brain barrier leakage rate and spatial extent: Considerations for dynamic contrast-enhanced MRI. Med Phys.

[73] Boschi F., Marzola P., Sandri M., Nicolato E., Galie M., Fiorini S. (2008). Tumor microvasculature observed using different contrast agents: a comparison between Gd-DTPA-Albumin and B-22956/1 in an experimental model of mammary carcinoma. MAGMA.

[74] Notohamiprodjo M., Pedersen M., Glaser C., Helck A.D., Lodemann K.P., Jespersen B. (2011). Comparison of Gd-DTPA and Gd-BOPTA for studying renal perfusion and filtration. J Magn Reson Imaging.

[75] Richardson O.C., Bane O., Scott M.L., Tanner S.F., Waterton J.C., Sourbron S.P. (2015). Gadofosveset-based biomarker of tissue albumin concentration: Technical validation in vitro and feasibility in vivo. Magn Reson Med.

[76] Thomsen H.S., Morcos S.K., Almen T., Bellin M.F., Bertolotto M., Bongartz G. (2013). Nephrogenic systemic fibrosis and gadolinium-based contrast media: Updated ESUR Contrast Medium Safety Committee guidelines. Eur Radiol.

[77] EMA (2017). EMA's final opinion confirms restrictions on use of linear gadolinium agents in body scans (EMA/457616/2017).

[78] FDA (2017). FDA Drug Safety Communication: FDA warns that gadolinium-based contrast agents (GBCAs) are retained in the body; requires new class warnings (12-19-2017).

[79] Gulani V., Calamante F., Shellock F.G., Kanal E., Reeder S.B., on behalf of the International Society for Magnetic Resonance in Medicine (2017). Gadolinium deposition in the brain: summary of evidence and recommendations. Lancet Neurol.

[80] Jenkinson M., Bannister P., Brady M., Smith S. (2002). Improved optimization for the robust and accurate linear registration and motion correction of brain images. Neuroimage.

[81] Akbudak E., Conturo T.E. (1996). Arterial input functions from MR phase imaging. Magn Reson Med.

[82] Korporaal J.G., van den Berg C.A., van Osch M.J., Groenendaal G., van Vulpen M., van der Heide U.A. (2011). Phase-based arterial input function measurements in the femoral arteries for quantification of dynamic contrast-enhanced (DCE) MRI and comparison with DCE-CT. Magn Reson Med.

[83] Sourbron S.P., Buckley D.L. (2012). Tracer kinetic modelling in MRI: Estimating perfusion and capillary permeability. Phys Med Biol.

[84] Brix G., Zwick S., Kiessling F., Griebel J. (2009). Pharmacokinetic analysis of tissue microcirculation using nested models: Multimodel inference and parameter identifiability. Med Phys.

[85] Ingrisch M., Sourbron S., Reiser M.F., Peller M. (2010). Model selection in dynamic contrast enhanced MRI: The Akaike Information Criterion. Ifmbe Proc.

[86] Paudyal R., Poptani H., Cai K., Zhou R., Glickson J.D. (2013). Impact of transvascular and cellular-interstitial water exchange on dynamic contrast-enhanced magnetic resonance imaging estimates of blood to tissue transfer constant and blood plasma volume. J Magn Reson Imaging.

[87] Ingrisch M., Sourbron S., Morhard D., Ertl-Wagner B., Kumpfel T., Hohlfeld R. (2012). Quantification of perfusion and permeability in multiple sclerosis: dynamic contrast-enhanced MRI in 3D at 3T. Invest Radiol.

[88] Parker G.J., Roberts C., Macdonald A., Buonaccorsi G.A., Cheung S., Buckley D.L. (2006). Experimentally-derived functional form for a population-averaged high-temporal-resolution arterial input function for dynamic contrast-enhanced MRI. Magn Reson Med.

[89] Tofts P.S., Kermode A.G. (1991). Measurement of the blood-brain barrier permeability and leakage space using dynamic MR imaging. 1. Fundamental concepts. Magn Reson Med.

[90] Weinmann H.J., Laniado M., Mutzel W. (1984). Pharmacokinetics of GdDTPA/dimeglumine after intravenous injection into healthy volunteers. Physiol Chem Phys Med NMR.

[91] Lavini C., Verhoeff J.J. (2010). Reproducibility of the gadolinium concentration measurements and of the fitting parameters of the vascular input function in the superior sagittal sinus in a patient population. Magn Reson Imaging.

[92] Tropres I., Pannetier N., Grand S., Lemasson B., Moisan A., Peoc'h M. (2015). Imaging the microvessel caliber and density: Principles and applications of microvascular MRI. Magn Reson Med.

[93] Tofts P.S., Brix G., Buckley D.L., Evelhoch J.L., Henderson E., Knopp M.V. (1999). Estimating kinetic parameters from dynamic contrast-enhanced T(1)-weighted MRI of a diffusable tracer: Standardized quantities and symbols. J Magn Reson Imaging.

